# Weight Loss-associated Superior Mesenteric Artery Syndrome, an Unusual Condition in a Peruvian Patient: A Case Report

**DOI:** 10.7759/cureus.3739

**Published:** 2018-12-17

**Authors:** Luis A Chirinos, Danitza Lukac, Mateo E Garland, Jorge André Céspedes, Victor M Salcedo

**Affiliations:** 1 Facultad De Medicina Humana, Universidad Científica Del Sur, Lima, PER; 2 Facultad de Medicina Humana, Universidad San Martin de Porres, Lima, PER; 3 Medicina Interna, Hospital Central De La Fap “comandante Fap Médico Juan Benavidez Dorich”, Lima, PER

**Keywords:** superior mesenteric artery syndrome, weight loss, small bowel obstruction, peru

## Abstract

Superior mesenteric artery syndrome (SMAS) is an uncommon cause of small bowel obstruction. We report a young male patient with abdominal pain, emesis and history of significant weight loss. Computed tomography angiography demonstrated narrowing of the aortomesenteric angle. The patient underwent conservative medical management, focusing on relieving obstruction and nutritional support.

## Introduction

Superior mesenteric artery syndrome (SMAS) is an uncommon cause of small bowel obstruction [1]. It is caused by vascular compression of the third part of the duodenum between the abdominal aorta and overlying superior mesenteric artery (SMA) due to an acute angle formation [2]. Several factors are associated with the narrowing of the aortomesenteric angle [3]. Usual clinical presentation consists of progressive history of general complaints like bloating, nausea, vomiting, postprandial epigastric pain, and other non-specific symptoms. In most cases it is a diagnosis of exclusion [4]. Imaging studies are considered essential for diagnosis. Computed tomography (CT) and angiography are widely used, although other studies are proven to be useful but with lesser sensitivity and specificity [5]. Treatment options range from conservative measures to surgical procedures depending on etiology, the evolution and severity of clinical presentation.

## Case presentation

A 19-year-old male patient with no significant past medical history presented to the emergency room (ER) at the Peruvian Air Force Hospital. He had been experiencing four hours of abdominal pain and emesis. He added that he had been on a strict regimen of dieting and exercise and had lost significant weight in the past months. Symptoms started after heavy meal ingestion and consisted of abdominal oppressive pain and distention accompanied by nauseas and bilious emesis. The family member stated the patient was undergoing a strict carbohydrate restriction, increase in daily exercise and had lost 25 kg in seven months.

On physical examination, the patient adopted dorsal decubitus position with knees flexed towards chest. Abdominal examination revealed scaphoid abdomen, diminished high-pitched bowel sounds and tender abdomen in mesogastrium and epigastrium regions. Laboratory workup was within normal limits. The patient stayed overnight in the ER and was managed conservatively with Nil per os (NPO), intravenous crystalloid rehydration, nasogastric tube, ondansetron and omeprazole. Next day reevaluation showed persistence of abdominal pain, nausea and bilious emesis.

**Figure 1:**
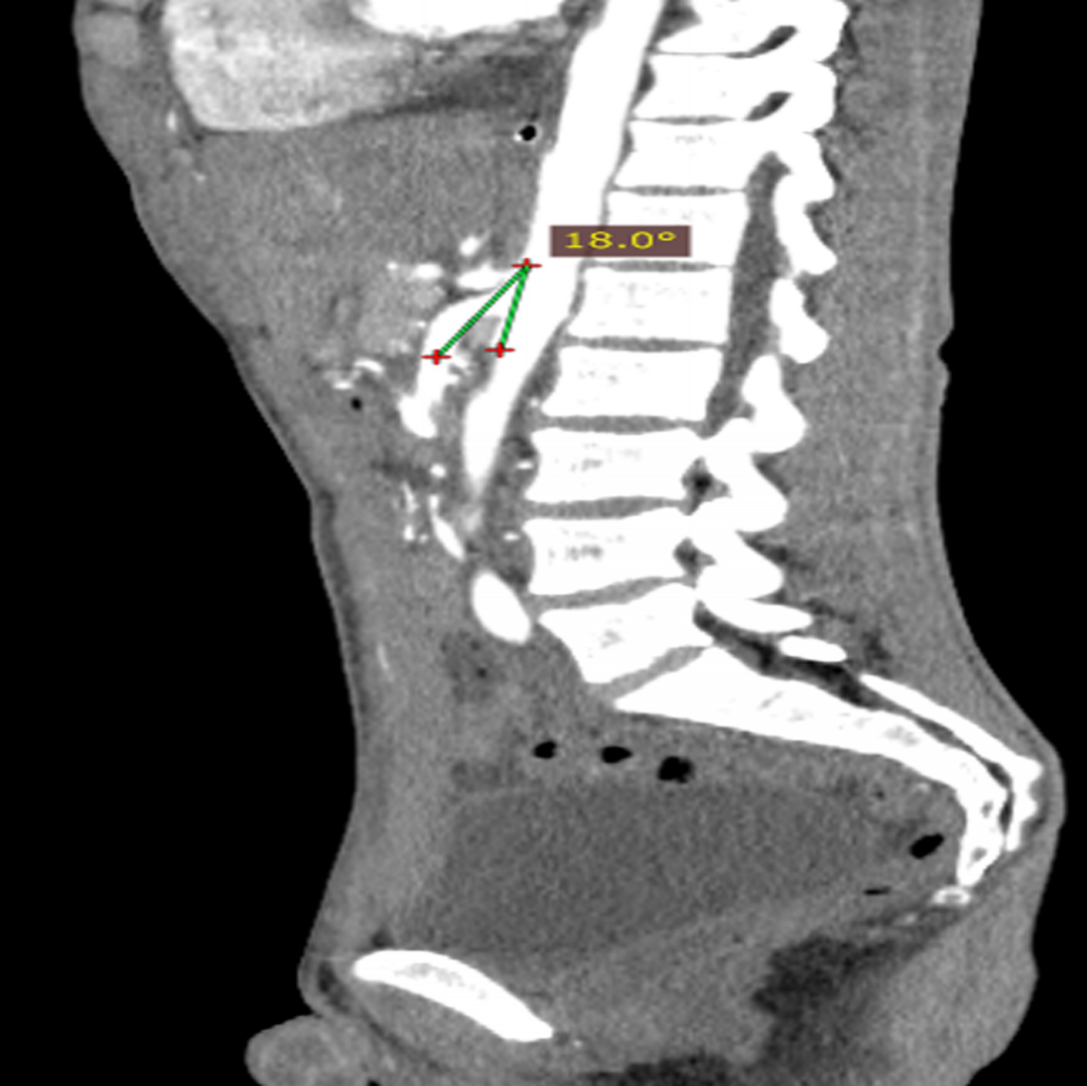
Sagittal computed tomography angiography (CTA) of the abdomen demonstrates a narrow aortomesenteric angle (18º).

**Figure 2:**
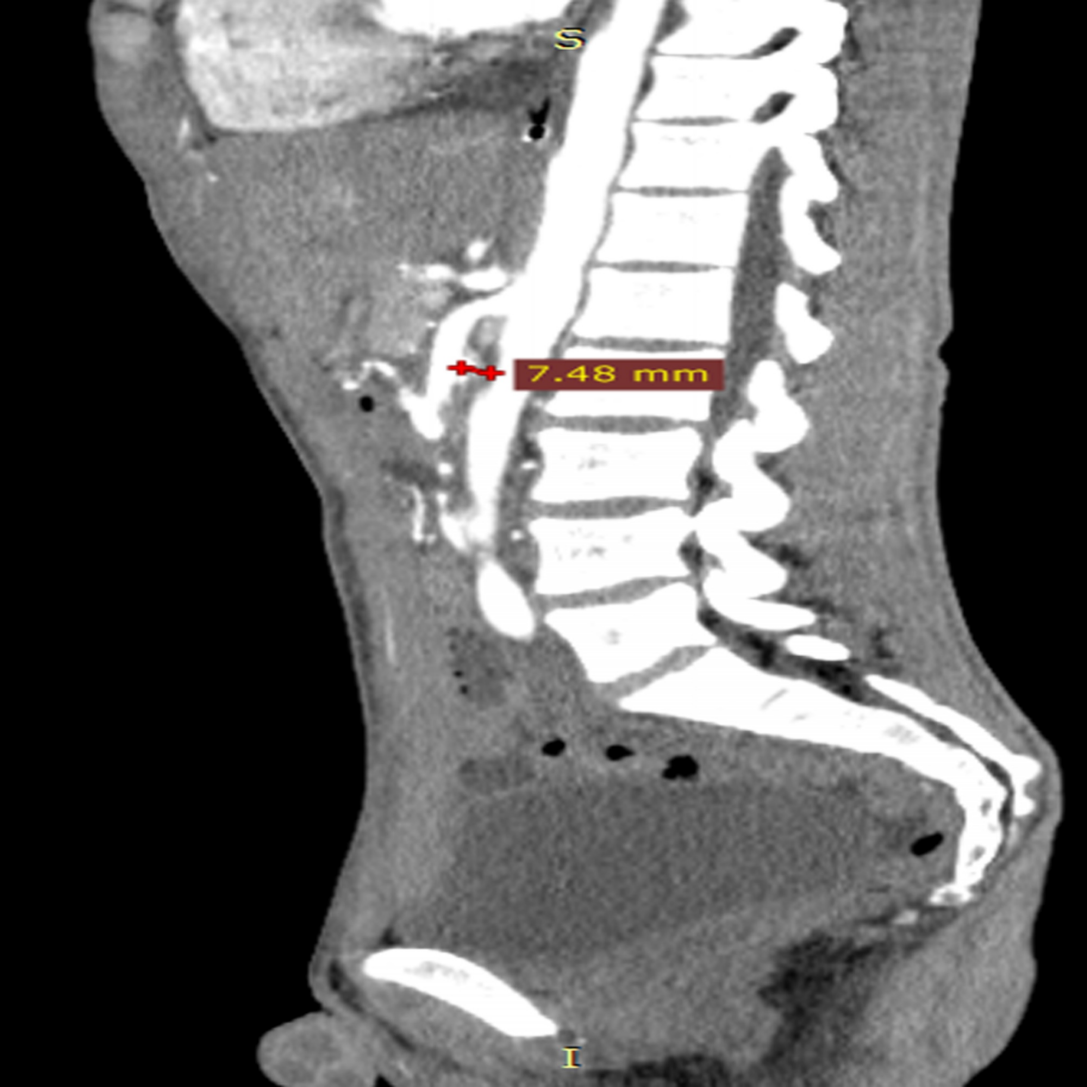
Sagittal computed tomography angiography (CTA) of the abdomen demonstrates a distance of 7.48 mm between the superior mesenteric artery and the aortic artery.

Computed tomography study revealed gastric dilatation extended to the third portion of the duodenum, suggestive of proximal small bowel obstruction. The patient was admitted for inpatient treatment. After general surgery consultation, he underwent computed tomography angiography (CTA) which found a narrow aortomesenteric angle 18° (Figure 1) and a distance between these structures of 7.48 mm (Figure 2).

Studies confirmed the diagnosis of small bowel obstruction due to SMAS. Initial management included gastric decompression by placing a nasojejunal feeding tube to alleviate symptoms of obstruction and later use for enteral nutritional support. A normocaloric hyperproteic formula was used starting with 600 and progressed to 1500 kcal per day to reverse protein caloric malnutrition state. Psychology and psychiatry departments were consulted concerning major weight loss. The patient was diagnosed with depression and an eating disorder. Sertraline was added to his treatment. The patient was discharged asymptomatic, with hyperproteic diet plus nutritional supplements and selective serotonin reuptake inhibitor. The patient's follow-ups showed weight gain and no symptoms. Psychiatry and psychology teams will follow up the patient after discharge.

## Discussion

SMAS is a rare cause of upper intestinal obstruction, reported for the first time by Von Rokitansky as an autopsy finding. However, the pathophysiology was described in 1927 by Wilkie, being his name frequently ascribed to this condition. This condition consists of extrinsic vascular compression and obstruction of the third portion of the duodenum between the superior mesenteric artery and the aorta [6, 7].

Estimated prevalence varies between 0.1 and 2.4% according to some case reports; lack of data makes prevalence still uncertain [8, 9]. This condition can occur at any age, although it is more common in females and young adults between 18 and 35 years old [6, 7].

There are no specific signs or symptoms suggestive of SMAS, making clinical diagnosis challenging and it is typically one of exclusion [10, 11]. The most relevant clinical findings include intermittent or postprandial abdominal pain, epigastric fullness, nausea, vomiting (sometimes bilious) and weight loss [1, 2].

Etiology of this condition is varied and can be divided into congenital and acquired [3]. Congenital factors include a low origin of the SMA or an abnormal high origin of Treitz ligament [12]. Acquired factors are numerous, they include hypercatabolism situations (burns, multiple trauma), surgical causes (bariatric surgery, corrective spinal surgery, esophagectomy, abdominal trauma), eating disorders (malabsorption, anorexia nervosa), and clinical conditions (AIDS, cerebral palsy, drug abuse, neoplasms). Previously-mentioned risk factors cause reduced thickness of the retroperitoneal adipose tissue and a diminished aortomesenteric space and angle [3, 10].

Differential diagnosis includes intraluminal intestinal obstruction, gastroesophageal reflux disease (GERD), functional dyspepsia, gastroparesis associated with metabolic disorders, neurogenic bowel disfunction associated with spinal injury (SCI), megaduodenum, chronic gastritis, diverticulitis, gastroduodenal ulcers and hiatus hernia [1, 6, 12].

Imaging studies are essential for the diagnosis. Conventional barium studies show findings compatible with small bowel obstruction such as dilation of the first and second portion of the duodenum with narrowing of the third portion with or without gastric dilatation. However, these signs can be seen on other causes of obstruction and are not pathognomonic [1]. Computed tomography and angiography have become the most widely used diagnostic methods, showing accurate measurements of the aortomesenteric angle and displaying the external compression of the duodenum [5, 13].

Anatomical parameters formed by the aorta and the SMA are the aortomesenteric angle which normally vary between 25º and 65º and the aortomesenteric distance of 10-28 mm [10, 14]. According to most studies, an aortomesenteric angle less than 22º and distance less than 8-10 mm are defined as imaging criteria for SMAS diagnosis [5, 15].

Conservative medical treatment is the first approach which includes fluid-electrolyte imbalance correction, gastric decompression, nutritional support through total parenteral nutrition or post-pyloric tube feeding by nasojejunal tube. Conservative treatment aims to restore retroperitoneal fat and weight gain. Indirect calorimetry is considered gold standard to measure caloric needs in these patients, but on the downside, it requires trained personnel, it is expensive and not readily available. The Harris-Benedict formula calculates basal metabolic rate, also considered as an option to measure caloric needs and is more commonly used [3, 16].

Surgical procedures are considered when conservative treatment fails [10]. Duodenojejunostomy is the procedure of choice, the success rate has been shown between 80% and 100%, where low rate of postoperative adhesions and complications are seen [9]. Other treatment options include division of the Treitz ligament with mobilization of the duodenum called Strong's procedure. Also, gastrojejunostomy is another option but is associated with postoperative complications like blind loop syndrome and recurrence of symptoms [11, 17].

## Conclusions

Superior mesenteric artery syndrome is an uncommon cause of small bowel obstruction. Most clinicians are not aware of this condition because of its low prevalence which delays the diagnosis. Clinical presentation and patient history are the keys to diagnosis, especially in cases of considerable weight loss and postprandial vomiting. Imaging support is essential for diagnosis confirmation. Medical conservative treatment is the first approach for management; when it fails surgery should be considered.
